# A portrait of anorexia nervosa

**DOI:** 10.1192/bjb.2021.92

**Published:** 2022-04

**Authors:** Lily Aston

**Affiliations:** Student, Eastbourne, UK

**Keywords:** Art, interpretation, anorexia nervosa, mental disorders, emotions

## Abstract

The author draws comparisons between the subjectivity of interpreting a work of art and that of experiencing anorexia nervosa. She explores her own experiences of anorexia through a portrait depicting the duality of the disorder.

What I like most about art is that it enables me to express complicated thoughts and emotions that often feel impossible to communicate verbally. Not only is it expressive, it is also fluid and its effect on others is completely subjective. It has no concrete rules, methods or regulations. It is completely versatile, and for a sufferer of anorexia nervosa, that freedom is certainly relieving. Defining my disorder for others has often been difficult for me as I find it cannot be categorised by a standardised list of reasons or symptoms. I believe it parallels art in that it is subjective to an individual, it has so many loop-holes and complexities. And so with that, my disorder will be different to the girl who sits opposite me in class or the boy who goes to my gym. We are all dealing with our own specialised disorder and that is something I wanted to express in my artwork.

## ‘It's all inside your head’

**Fig. 1:**
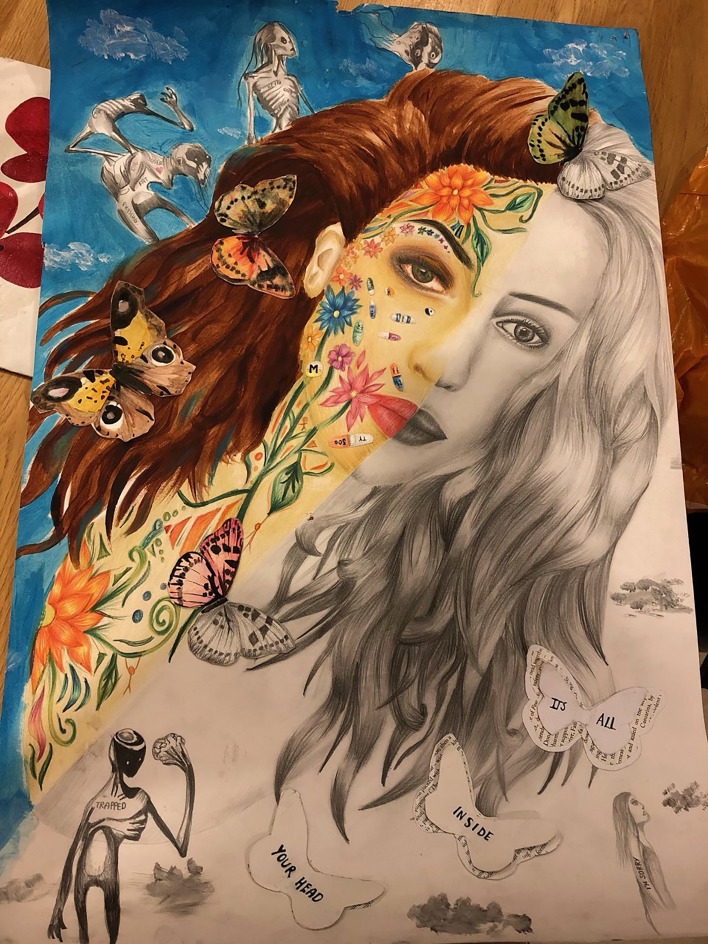
*It's all inside your head* (2020). Lily Aston.

Since finishing this piece (Fig. 1) I have listened to a lot of people's interpretations of it and I love how everyone came up with a different approach to what it represents. The two sides of the portrait are meant to represent what is visible of an individual with anorexia and then the complexities that lie below the surface. While creating ideas for the visible side of the portrait I thought about what a typical sufferer of anorexia is perceived to look like. As a society we automatically assume the individual to be underweight, malnourished, pale and quite frankly on the cusp between life and death. Unfortunately, statistical research will show that in a lot of cases these features are disturbingly common. But I have always held on to the belief that these visible symptoms are not the determining aspects that separate a sufferer from a non-sufferer. When I asked my Mum about the time I was in the deep throes of anorexia she said something that has stayed with me throughout my recovery. She simply told me ‘there was no one there’. That idea of an absence of self being a major symptom of anorexia is definitely something I believe fellow sufferers will identify with. The defensive remarks, spiteful comments and the extreme memory loss are all evidence that the person I knew I was, was no longer present. A lot of sufferers will personify their illness to detach it from who they are as an individual. In the case of anorexia, this person is commonly referred to as ‘Ana’ and this double identity was an important idea that I wanted to feature in my artwork.

My personal interpretation of the piece is that the black and white side represents this ‘absence of self’. The lack of colour and and detail establishes the loss of personality, it is what others may see when they face an anorexia sufferer. The coloured side then represents what lies below the surface, the complexity of emotions: confusion, anger, sadness, resentment. These all combine to create this explosion of chaotic colour. They are the emotions that sufferers will tend to hide from the outside world in order to remain ‘undercover’. That is why many victims of anorexia will go through a major period of denial, convincing others and quite often themselves that they are ‘fine’, ‘I couldn't be happier’, ‘I feel so much healthier now’, ‘oh no thank you, I'm not hungry’. Humans are emotionally complex beings and expression of these emotions is necessary to maintain our sanity. So in the case of an anorexia victim going through denial, it is only a matter of time before this wall is broken and these emotions come flooding out, quite often with a vengeance. I remember very clearly when my wall broke down. I came home from school and went straight to my room to lie on my bed. I had eaten about 600 calories worth of food in the span of three days and by that point my body was too weak to feel the hunger pains. I lay there for a couple of hours until my Mum called me down to say ‘We're going to order a takeaway’. And I was – furious. My perfect utopian façade had loosened at the seams and all this hate, fury and fear came bursting out in all directions as I screamed at these people who I loved more than anything in the world. I am ashamed to admit that in that moment I cursed their existence and wished they would disappear.

## Unlocking the chain

That evening was a turning point for me, it was as if my double identity had begun to reveal itself to those around me, including myself. And it was important for me to convey this duality in my portrait. I considered doing two different faces as I hated the idea of anorexia being a part of me. But the harsh reality is that it doesn't become a part of you – it overcomes you. Recovering from an eating disorder is like peeling a hundred layers of your skin from your body. It is extremely painful, both physically and mentally. But this shedding of skin is absolutely essential in order to detach yourself from the grip of anorexia. Throughout recovery, it is so important to realise that an eating disorder does not become a permanent part of your skin. I view it as a locked chain that is wrapped around your body, constricting your movements. It feels impossible to escape and your situation feels hopeless for quite a long time. But it is through deep inner strength and the support of others that we are made to realise that we had the key in our back pockets all along. However, recovery is not as brief and straightforward as unlocking a chain. Our chains are quite often tangled and complicated. It takes a lot of time and patience. But I say from experience, it is a challenge worth taking.

## The journey of creation and the journey of recovery

The process of making this piece of art was a long and tough journey but I hope others who are currently battling with their own demons will be able to identify with it. I welcome and encourage people to interpret it however they like. For all our journeys are different in their many complexities, but I hope we will all end up at the same destination. Content and healthy.

I must also mention an incredible artist called Shawn Coss, whose interpretations of mental disorders truly touched and inspired me to create this piece. The haunting figures that are scattered around the portrait are features of his work. His talent for expressing such complex emotions encouraged me to express my own, and I hope that others will be inspired to do the same.

## Data Availability

Data availability is not applicable to this article as no new data were created or analysed in its writing.

## About the author

**Lily Aston** is a student currently on a gap year. Due to study Psychology at Bristol University in 2022 but currently resides in Eastbourne, UK.

